# Socioeconomic inequalities in adolescent health complaints: A multilevel latent class analysis in 45 countries

**DOI:** 10.1007/s12144-022-03038-6

**Published:** 2022-04-01

**Authors:** Nour Hammami, Inese Gobina, Justė Lukoševičiūtė, Michaela Kostičová, Nelli Lyyra, Genevieve Gariepy, Kastytis Šmigelskas, Adriana Baban, Marta Malinowska-Cieślik, Frank J. Elgar

**Affiliations:** 1 Institute for Health and Social Policy, McGill University, 1130 Pine Avenue West, Montreal, Quebec H3A1A3 Canada; 2 Johnson-Shoyama Graduate School of Public Policy, University of Regina, Regina, Saskatchewan Canada; 3 Faculty of Public Health and Social Welfare, Department of Public Health and Epidemiology, Riga Stradiņš University, Rīga, Latvia; 4 Health Research Institute, Faculty of Public Health, Medical Academy, Lithuanian University of Health Sciences, Kaunas, Lithuania; 5 Institute of Social Medicine and Medical Ethics, Faculty of Medicine, Comenius University in Bratislava, Bratislava, Slovakia; 6 Faculty of Sport and Health Sciences, University of Jyväskylä, Jyväskylä, Finland; 7 Public Health Agency of Canada, Ottawa, Canada; 8 Department of Health Psychology, Lithuanian University of Health Sciences, Kaunas, Lithuania; 9 Department of Psychology, Babes-Bolyai University, Cluj-Napoca, Romania; 10 Department of Environmental Health, Faculty of Health Sciences, Jagiellonian University Medical College, Kraków, Poland

**Keywords:** Multiple health complaints, Psychosomatic, Mental health, Socioeconomic position, Adolescents, HBSC

## Abstract

Our study evaluated the relationship between adolescent health complaints and socioeconomic position in 45 countries. Data are from the 2017/2018 international Health Behaviour in School-aged Children survey which used proportionate sampling among adolescents aged 11 to 15 years old (*n*=228,979). Multilevel, multinomial regression analysis assessed the association between the multilevel latent classes with socioeconomic status (SES; at the household and country level). Three distinct latent classes were identified: No Complaints, Psychological Complaints, and a Physical and Psychological Complaints class; where, low household SES was highest for the physical and psychological complaints class. The findings suggest that health promotion policies and interventions among adolescents should consider the specific needs of adolescents living with low household SES as they report more subjective health complaints.

## Background

Adolescence is often considered the healthiest time of life (Patton et al. 2016); yet, a recent report finds that 35% of adolescents experience multiple psychosomatic health complaints in Canada and European countries (Inchley et al. 2020). Subjective health complaints (i.e., psychosomatic complaints) are self-reported health complaints that often have no obvious organic cause and are often viewed as a stress-reaction to psychosocial tensions (Ravens-Sieberer et al. 2009). According to the shared vulnerability model, physical pain is associated with anxiety disorders and other negative emotional responses among adolescents and adults (Asmundson and Katz 2009; Jastrowski Mano et al. 2019). For adolescents, the shared vulnerability model (Jastrowski Mano et al. 2019) suggests that the development and maintenance of chronic pain and poor mental health have shared individual predisposing factors such as stressors, traumatic events, vulnerabilities, and low threshold for alarm. Increased sensitivity to stress gives rise to negative emotional and cognitive responses, such as catastrophizing, fear, and anxiety. These responses ensure the maintenance of pain and poor mental health by increasing avoidance behaviours, cognitive biases, and autonomic nervous system arousal – ultimately resulting in a disabling condition of chronic pain and anxiety (Jastrowski Mano et al. 2019). Health complaints can negatively impact adolescents’ daily life, functional status, and mental health (Potrebny et al. 2017).

Many factors are associated with multiple health complaints, including behavioral, demographic, environmental, and socioeconomic factors (Ravens-Sieberer et al. 2009; Ottová-Jordan et al. 2015; Vaičiūnas and Šmigelskas 2019). Namely, socioeconomic inequalities are recognized as important determinants of health and well-being among the general population (Marmot 2004; Wilkinson and Pickett 2009) and among adolescents (Currie et al. 2012; Chzhen et al. 2016). Early research in health complaints found that a lower socioeconomic status (SES) was associated with higher odds of health complaints (Ravens-Sieberer et al. 2009) and that both relative and absolute differences in household SES accounted for a significant variation in adolescent health complaints (Elgar et al. 2013). The self-determination theory postulates that ongoing psychological growth, integrity, and wellbeing can be achieved when basic psychological needs are met (Deci and Ryan 2000). The theory defines basic psychological needs as feelings of autonomy, competence, and relatedness or social connectedness (Deci and Ryan 2000). Di Domenico and Fournier (2014) built upon these ideas by examining whether the association of SES and health complaints were moderated or mediated by basic psychological needs. Subjective SES and objective household SES were positively associated with greater need for fulfillment, which in turn was negatively associated with health complaints. These findings suggest that low individual and household SES are associated with more health complaints, possibly through unmet basic psychological needs.

Further, macro-level socioeconomic factors, such as national income inequality and national wealth, are associated with negative health outcomes (Wilkinson and Pickett 2009). While early past evidence finds that variations in health complaints among adolescents are mostly explained by individual-level (not macro-level) SES (Holstein et al. 2009; Ottová-Jordan et al. 2015), investigations in the current socioeconomic context are warranted. Given the rising prevalence of health complaints among adolescents, an up to date, in-depth analysis is needed to investigate the association between multiple health complaints and socioeconomic inequalities.

Evidence on adolescent health complaints has also been informed by modelling health complaints’ data as a single continuous score (Ravens-Sieberer et al. 2008; Cosma et al. 2020). However, health complaints are not a continuous scale but distinct and correlated physical and psychological health symptoms (Hetland et al. 2002; Gariepy et al. 2016). Measuring health complaints on a continuous scale limits our understanding of the nature of the complaints and their associations with social and health measures. For example, the number of complaints might be the same for a certain group, but the type and severity of the complaints may vary across that group. The extent to which individuals with similar complaints may have similar health and social determinants or outcomes are independent of the number of complaints reported. Thus, novel methodological approaches in measuring psychosomatic complaints that take into consideration the nature and co-occurrence of complaints are warranted.

A novel approach when assessing associations between several correlated variables is to identify which are experienced together and to statistically identify underlying (latent) classes of these variables in a data-driven manner. Latent class analysis (LCA) is a person-centred approach to investigating sets of characteristics and experiences (Lanza et al. 2007). The approach addresses a common challenge in health research where multiple overlapping predictors of a health outcome each explain a small amount of variation in an outcome (Collins and Lanza 2010). Therefore, mutually controlled associations are not necessarily of most import, nor do they resemble the natural clustering of risk and protective factors in real-world settings. Clinical and policy interventions rarely require evidence on singular statistical associations between specific variables.

Often, interventions require evidence about profiles of risk and resilience while adjusting for correlations between adolescents from the same area or location. This can be achieved using a multilevel latent class analysis (MLCA) which identifies which complaints make up each latent class (Henry and Muthén 2010). MLCA shows how adolescents experience multiple health complaints in their daily life, which tend to co-occur and how many distinct classes can be identified. An advantage of the MLCA is that it takes accounts for the clustered nature of the data which is especially important in international investigations (Henry and Muthén 2010). Then, these latent classes can be used as a dependent variable and associations can be drawn with other factors.

## The study’s aims and hypotheses

Drawing from the shared vulnerability model (Asmundson and Katz 2009; Jastrowski Mano et al. 2019), the self-determination theory (Deci and Ryan 2000), Di Domenico and Fournier (Di Domenico and Fournier 2014), and co-occurrence methodology (Lanza et al. 2007; Henry and Muthén 2010), this study examined (1) the co-occurrence of patterns of psychosomatic health complaints among adolescents by identifying latent classes of health complaints and (2) the relationship between the latent classes of multiple health complaints with socioeconomic status among adolescents from 45 countries participating in 2017/2018 HBSC survey. Based on the literature, we hypothesized that (1) physical pains and psychological complaints co-occured (Jastrowski Mano et al. 2019), possibly in the same latent classes. We also hypothesized that (2) adolescents in classes with more health complaints were more prevalent at lower socioeconomic status, in less wealthy countries, and more unequal in countries with more income inequality (Marmot 2004; Wilkinson and Pickett 2009).

## Methods

### The Host Study: Health Behaviour in School-aged Children (HBSC) survey

Data are from the 2017/2018 international Health Behaviour in School-aged Children (HBSC) survey. HBSC is a school-based survey that is carried out every four years in collaboration with the World Health Organization Regional Office for Europe and a host of researchers across 45 countries in Europe and North America. The HBSC aims to investigate adolescents’ health and well-being (subjective health, health complaints, life satisfaction), social environments (family, peers, school environment), and health behaviours (tobacco, alcohol, cannabis, drug use) across Europe and North America. This international questionnaire allows for national and cross-national investigations and promotes the collaboration of multidisciplinary teams of researchers across the respective countries.

### Survey administration

Adolescents filled the surveys during school hours, and it took approximately 45 minutes to complete. The data were collected by following the international data protocol (Inchley et al. 2020). Ethical approval was granted by the appropriate country or region from a university-based review board or equivalent body. Surveys were administered by researchers or teachers using a standard protocol. Standardized information about the study were provided to adolescents and their caregivers. Either active-consent or passive-consent approaches depending on country and school board requirements were used. In addition to flexible withdrawal procedures, surveys were collected in adolescents’ respective classrooms during school hours with their teacher or an HBSC researcher present to provide a secure experience. For representative sampling, probability proportionate to size sampling of adolescents at schools was used. Additional details on the HBSC survey design and development are available elsewhere (Roberts et al. 2007, 2009; Inchley et al. 2020).

### Participants

In each country, representative samples of 11-, 13-, and 15-year-old adolescents from general schools were selected. In our analyses, a total of 45 countries and regions across Europe and North America were included, comprising 1,008 schools and 228,979 adolescents aged 11-15 years old. The mean age was 13.5 years old (SD=1.6), and the sample was comprised of 50.6% female, and 49.4% males (the option for “non-gender binary” was not available across all countries participating in the international survey).

### Measures

#### Multiple health complaints checklist (dependent variable)

The HBSC measures multiple health complaints using a checklist which assesses the following physical and psychological complaints: headache, abdominal pain, backache, dizziness, feeling low, irritability or bad mood, feeling nervous, and sleeping difficulties. The scale has proven validity across countries (Haugland and Wold 2001; Ravens-Sieberer et al. 2009; Hagquist et al. 2019).

#### Micro-level socioeconomic status (independent variable)

Household SES was measured using the HBSC Family Affluence Scale (FAS), a 6-item measure of common material assets in the home (Currie et al. 2008): “Do you have your own bedroom for yourself?”, “How many bathrooms are in your home?”, “Does your family own a car, van or truck?”, “How many times did you and your family travel out of your country for a holiday last year? ”, “Does your family have a dishwasher at home?” and “How many computers does your family own?”. FAS has been found to be a valid measure of adolescent SES across several countries (Molcho et al. 2007; Hobza et al. 2017; Corell et al. 2021).

#### Macro-level socioeconomic status (independent variables)

We included two macro-level variables that are strong determinants of adolescent health: national wealth and income inequality (Dorling et al. 2007; Swift 2011; Ward and Viner 2017). For national wealth, we used national gross domestic product per capita (in trillions of current United States dollars), provided by the World Bank from 2018 (The World Bank 2018). As for income inequality, we used 2017’s National Gini coefficients from the Standardized World Income Inequality Database (Solt 2016).

#### Control variables (independent variables)

The control variables in the model were: gender, age, family meals together, drunkenness, tobacco smoking, and cannabis use. These have been reported to be associated with the socioeconomic status gradient as well as with health complaints (Elgar et al. 2013; Coley et al. 2018).

### Data analyses

#### Data management

##### Multiple health complaints checklist

The question in the HBSC survey asks “In the last 6 months, how often have you had the following? (Please mark one box for each line)” with each line containing one of the complaints. Response categories included: about every day, more than once per week, about every week, about every month or rarely/never. Each psychological and physical complaints was dichotomized in our analyses with adolescents either having: (i) frequent (more than once a week) or (ii) infrequent (once a week or less) complaints. This was done in-line with previous HBSC research (Inchley et al. 2020) and for simpler interpretation of the latent classes (Henry and Muthén 2010; Hammami et al. 2019).

##### Micro-level socioeconomic status: Household SES (independent variable)

A total FAS score was calculated (ranging from 0 to 13). It was then harmonized by transforming the total score on the FAS scale to weighted proportional ranks (ridit scores) (Mackenbach and Kunst, 1997; Elgar et al. 2017). This resulted in a SES index (of material deprivation, or slope index of inequality) that ranged from 0 (lowest household SES, or the most deprived) to 1 (highest household SES, or the least deprived) , with a mean of 0.5 (Elgar et al. 2017). We then reversed the index to have 0 represent the highest household SES (i.e., least deprived) and 1 represent the adolescents living with the lowest SES (i.e., most deprived) (Elgar et al. 2017).

#### Summary statistics

Summary statistics for the multiple health complaint variables and covariates were reported as a percent and as frequencies.

#### Multilevel latent class analysis (MLCA)

MLCA has been described in detail by Henry and Muthén (2010); MLCA grouped the multiple health complaints into latent classes grouping the most co-occurring complaints into classes. In addition to identifying latent classes, MLCA considers the dependence of students within the same school, unlike the latent class analysis (LCA). For our analyses, 25 models were evaluated for model fit. The appropriate model was chosen after assessing: lowest Bayesian Information Criterion (BIC), highest entropy and the interpretability of the classes – as recommended and reported in previous research (Lanza et al. 2007; Henry and Muthén 2010; Hammami et al. 2019).

Previous MLCA analyses among adolescents show that MLCA classes are constant across gender, despite gender differences being observed across the adolescents’ behaviours that were included in the MLCA (Hammami et al. 2019); thus, our current analyses did not use a gender-specific MLCA. MLCA was conducted in Mplus (Muthén and Muthén 2018), while Stata 16.0 (Stata Press 2019) was used for all other analyses.

#### Regression analyses

We used mixed-effects, weighted, multinomial regression models to regress the multiple health complaints’ latent classes onto SES and the control variables. Models 1 and 3 show the odds of being in a latent class with psychological complaints versus no complaints, Models 2 and 4 show the odds of being in a latent class with psychological and physical complaints versus no complaints. All models accounted for the three-level clustered nature of the data at the level of the individual, school, and country.

## Results

### Participant characteristics

Table 1 shows summary statistics from this sample of adolescents. It displays the complaints in decreasing manner: feeling irritable (42.7%), feeling nervous (42.0%), sleep difficulties (34.9%), feeling low (30.4%), headaches (29.1%), backaches (21.6%), abdominal pain (19.5%) and feeling dizzy (17.5%).

**Table 1:** Summary statistics among adolescents participating in HBSC – International in 2017/8

	Percent	Frequency
Gender		
Females	50.6	115,762
Males	49.4	113,217
Total	100	228,979
Age (in years)		
11	34.2	56,645
13	33.8	56,715
15	32.0	52,347
Total	100	165,707
Irritable		
Frequent	42.7	95,164
Infrequent	57.3	127,456
Total	100	222,620
Nervous		
Frequent	42.0	93,444
Infrequent	58.0	129,264
Total	100	222,708
Sleep Difficulty		
Frequent	34.9	77,105
Infrequent	65.1	143,889
Total	100	220,994
Feel low		
Frequent	30.4	67,479
Infrequent	69.6	154,926
Total	100	222,405
Headache		
Frequent	29.1	65,393
Infrequent	70.9	159,559
Total	100	224,952
Backache		
Frequent	21.6	48,122
Infrequent	78.4	174,552
Total	100	222,674
Abdominal pain		
Frequent	19.5	43,694
Infrequent	80.5	179,869
Total	100	223,563
Dizzy		
Frequent	17.5	38,580
Infrequent	82.5	182,200
Total	100	220,780
Family meals together		
Every day	49.3	105,912
Most days	32.5	69,636
About once a week	8.8	18,785
Less often	6.8	14,605
Never	2.6	5,517
Total	100	214**,**455
Drunk behaviour in last 30 days		
No	93.8	200,360
Yes	6.2	12,990
Total	100	213,350
Smoked in last 30 days		
No	93.0	201,491
Yes	7.0	15,090
Total	100	216,581
Cannabis use in last 30 days		
No	94.4	90,997
Yes	5.6	5,290
Total	100	96,287

Frequent = more than once a week, infrequent = once a week or less.

### Multilevel latent class analysis (MLCA)

#### MLCA fit statistics

Table 2 shows the fit statistics for the 25 MLCA models including: the fixed effects models, random effects models, and random effects models with a continuous factor. The models with the common factor offered better fit statistics than the models without the common factor (judging by lower log-likelihood, BIC, and higher entropy).

**Table 2:** Fit statistics for the multilevel latent class analysis among adolescents participating in HBSC – International in 2017/8

	Number of student (level 1) latent classes			
	1	2	3	4
Fixed effects model				
Number of free parameters	8	17	26	35
Log-likelihood	-1047416.7	-929548.4	-915835.4	-910637.4
BIC	2094932.1	1859306.4	1831991.5	1821706.4
Entropy	0	0.742	0.695	0.642
Random effects nonparametric multilevel latent class analysis models				
2 school (level 2) latent classes				
Number of free parameters	9	19	29	39
Log-likelihood	-1047416.7	-929296.25	-915528.8	-910273.4
BIC	2094944.5	1858826.8	1831415.2	1821027.7
Entropy	0.124	0.849	0.797	0.746
3 school (level 2) latent classes				
Number of free parameters	10	21	32	43
Log-likelihood	-1047416.7	-929175.1	-915385.9	-910139.4
BIC	2094956.8	1858609.2	1831166.5	1820809.1
Entropy	0.329	0.761	0.737	0.702
4 school (level 2) latent classes				
Number of free parameters	11	23	35	47
Log-likelihood	-1047416.7	-929156.8	-915355.6	-91008.3
BIC	2094969.1	1858597.2	1831142.8	1820752.2
Entropy	0.009	0.785	0.691	0.702
Random effects nonparametric multilevel latent class analysis models with a continuous factor on level 1 latent class indicators				
2 school (level 2) latent classes				
Number of free parameters	-	30	42	54
Log-likelihood	-	-928205	-914776	-909526.2
BIC	-	1856779.9	1830070	1819718.4
Entropy	-	0.867	0.811	0.759
3 school (level 2) latent classes				
Number of free parameters	-	34	48	62
Log-likelihood	-	-928076	-914604.3	-909352.5
BIC	-	1856571.3	1829800.6	1819469.6
Entropy	-	0.753	0.746	0.783
4 school (level 2) latent classes				
Number of free parameters	-	38	54	70
Log-likelihood	-	-928025	-914486.9	-909283.9
BIC	-	1856518.5	1829639.7	1819431.1
Entropy	-	0.78	0.768	0.732

Among the models, the model that best described latent classes of multiple health complaints in our sample was the three school and three student latent classes and used a continuous factor, considering the interpretability of the classes. The fit statistics for this model were: 48 free parameters, -914604.3 Log-likelihood, 1829800.6 BIC, and 0.746 entropy. This model also offered a variety of school and student classes to reflect the heterogeneity of the sample, in line with previous research (Lanza et al. 2007; Henry and Muthén 2010; Hammami et al. 2019).

#### MLCA findings

The MLCA revealed three profiles of student latent classes: (1) the No Complaint group of adolescents who reported no health complaints (44.0% of adolescents), (2) the Psychological Complaints group with predominantly psychological complaints (43.1%) and (3) the Physical and Psychological Complaints group which reported both physical and psychological complaints (12.9%).

**Fig. 1:**
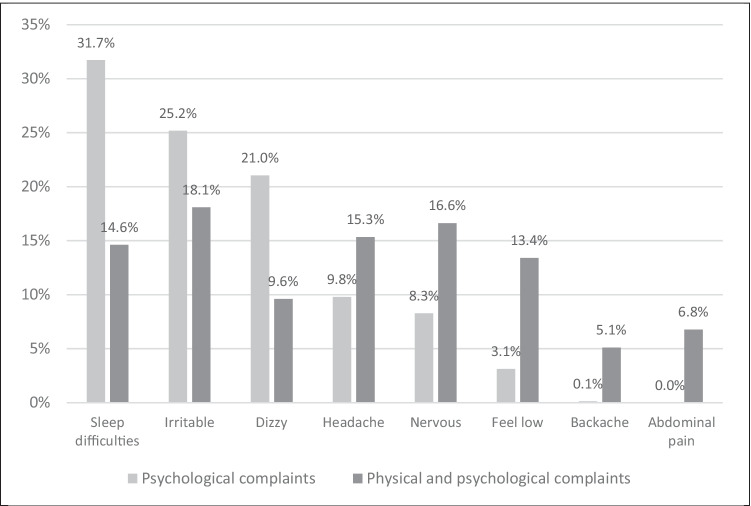
Distribution of the multiple health complaints in the latent classes from adolescents with complaints participating in HBSC- International in 2017/8.

Among those in the Psychological Complaints group, 31.7% complained of sleep difficulties, 25.2% of irritability and 21.0% of dizziness, while less than 10% complained of headaches (9.8%), nervousness (8.3%) and feeling low (3.1%) (Fig. 1). Among those in the Physical and Psychological Complaints group, 18.1% complained of irritability, 16.6% of nervousness, 15.3% of headaches, 14.6% of sleep difficulties, 13.4% of feeling low, 9.6% of dizziness, 6.8% of abdominal pain and 5.1% of backaches (Fig. 1). While both groups reported health complaints, adolescents in the Psychological Complaints group complained more specifically about three mostly psychological complaints, whereas adolescents in the Psychological and Physical Complaints group reported a low prevalence of a wide range of health complaints.

**Fig. 2:**
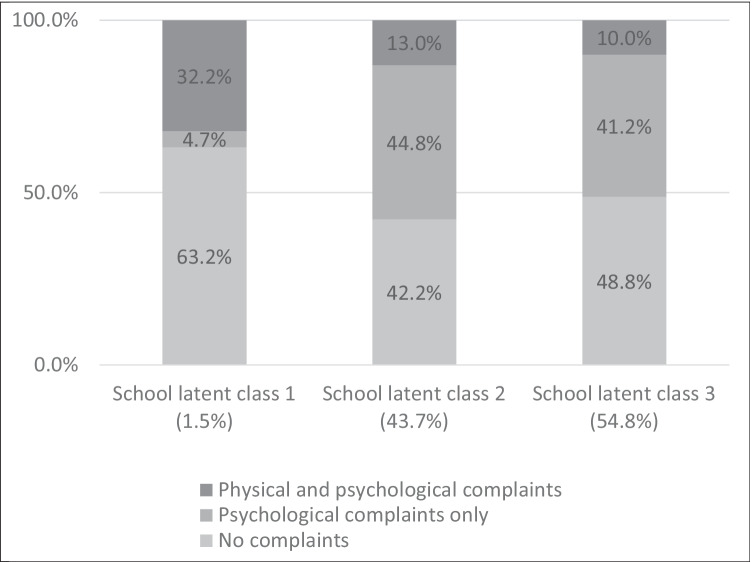
Distribution of student latent classes in school latent classes from the multilevel latent class analysis model from adolescents participating in HBSC- International in 2017/8.

Fig. 2 shows adolescent’s distribution in the three school and three student latent classes. The first school latent class consisted of 1.5% of the schools, the second 43.7% of the schools and the third consisted of 54.8% of schools. Although the first latent class of schools only consists of 1.5% of schools, these schools are important to identify since they have the highest proportion of students who belong to the no complaints latent class (63.2%) and the highest proportion of adolescents belonging to the physical and psychological complaints latent class (32.2%), relative to the other school latent classes, with the remaining 4.7% of adolescents in the psychological complaints latent class (See Fig. 2). School latent class 3 had the second highest proportion of adolescents belonging to the no complaints latent class (48.8%) and the lowest proportion of adolescents belonging to the physical and psychological complaints latent class (10.0%), relative to the other school latent classes, with the remaining 41.2% of adolescents in the psychological complaints latent class. As for school latent class 2, these schools had the lowest proportion of adolescents belonging to the no complaints latent class (42.2%) and the second from the highest proportion of adolescents belonging to the physical and psychological complaints latent class (13.0%), relative to the other school latent classes, and the highest proportion of adolescents in the psychological complaints latent class (44.8%). In line with our research question, the remainder of our analyses will address the adolescent latent classes – evaluating school latent class differences in adolescent health is an aspect for future research to explore.

### Regression analyses

Table 3 shows the regression models of the health complaints’ latent classes on household SES and control variables. Both national wealth and national income inequality were not significant in the Models 1 and 2; thus, country level variables are not found to be associated with latent classes of health complaints among adolescents in HBSC’s international sample in 2017/2018.

**Table 3:** Odds ratios (and 95% Confidence intervals) for being in latent classes with health complaints regressed on household socioeconomic status, country level variables and control variables, among HBSC adolescents participating in 2017/8 as part of the International sample.

	Model 1 ^a^	Model 2 ^b^	Model 3 ^a^	Model 4 ^b^
Household SES	1.16***	1.27***	1.14**	1.24***
	(1.08 - 1.24)	(1.15 - 1.40)	(1.05 - 1.24)	(1.11 - 1.37)
Gender				
Males (Ref.)				
Females	1.91***	4.35***	1.91***	4.29***
	(1.72 - 2.12)	(3.75 - 5.05)	(1.73 - 2.10)	(3.73 - 4.94)
National wealth, country level	1.03	1.06	-	-
	(0.94 - 1.14)	(0.91 - 1.24)	-	-
Income inequality, country level	0.18	0.23	-	-
	(0.0081 - 4.06)	(0.0013 - 40.1)	-	-
Age	1.11***	1.20***	1.11***	1.20***
	(1.09 - 1.14)	(1.13 - 1.28)	(1.09 - 1.14)	(1.13 - 1.28)
Family meals together				
Every day (Ref.)				
Most days	1.25***	1.51***	1.25***	1.49***
	(1.19 - 1.31)	(1.39 - 1.64)	(1.19 - 1.31)	(1.37 - 1.62)
About once a week	1.57***	2.39***	1.57***	2.36***
	(1.44 - 1.71)	(2.13 - 2.68)	(1.45 - 1.71)	(2.10 - 2.65)
Less often	1.83***	2.87***	1.85***	2.87***
	(1.67 - 2.01)	(2.51 - 3.27)	(1.69 - 2.02)	(2.53 - 3.26)
Never	2.31***	4.77***	2.34***	4.72***
	(1.96 - 2.73)	(4.11 - 5.53)	(1.20 - 2.73)	(4.06 - 5.48)
Drunk behaviour in last 30 days				
No (Ref.)				
Yes	1.30***	1.80***	1.30***	1.80***
	(1.22 - 1.38)	(1.66 - 1.96)	(1.22 - 1.38)	(1.65 - 1.95)
Smoked in last 30 days				
No (Ref.)				
Yes	1.64***	2.37***	1.64***	2.37***
	(1.49 - 1.80)	(2.09 - 2.68)	(1.50 - 1.79)	(2.09 - 2.69)
Cannabis use in last 30 days				
No (Ref.)				
Yes	1.24**	1.62***	1.26***	1.62***
	(1.08 - 1.42)	(1.38 - 1.89)	(1.10 - 1.43)	(1.39 - 1.90)

*** p<0.001, ** p<0.01, * p<0.05

^a^Outcome: Latent class with psychological complaints versus no complaints (Ref.)

^b^Outcome: Latent class with physical and psychological complaints versus no complaints (Ref.)

Table 3 also shows results from the models described above while omitting the macro-level variables (Models 3 and 4). Model 3’s results show that adolescents with the lowest household SES were 14% (Odds ratio [OR] =1.14, 95% Confidence Interval [C.I.] = 1.05, 1.24) more likely to belong to the Psychological Complaint group than the No complaint group. Model 4’s results show that adolescents with the lowest household SES were 24% (OR =1.24, 95% C.I. = 1.11, 1.37) more likely to belong to the Physical and Psychological complaints group than the No complaint group.

## Discussion

This study identified three different profiles of health complaints among adolescents across 45 countries and assessed their association with material deprivation. Our findings indicate that adolescents belong to one of three health complaint classes: no multiple health complaints, psychological complaints, and a combination of physical and psychological complaints. Low household SES was highest in the latent class with physical and psychological complaints. At the macro-level, the country level indicators national wealth and income inequality were not associated with latent classes of health complaints in our sample of HBSC international adolescents in 2017/2018.

A comprehensive approach to studying health complaints supports a better understanding and more-informed choices directed at preventing, treating, and managing their association with negative health outcomes in adolescence and later in life. Health complaints do not occur in isolation and profiles of health complaints may play key roles in the development of physical complaints, psychological complaints, and poor mental health outcomes. For example, it was recently reported that sleep-related problems partially mediated the association between irritability and anxiety (Poznanski et al. 2018). Our study also found that irritability co-occurred with sleep difficulties in both latent classes of adolescents. Another recent study found that adolescents with physical complaints (specifically migraines) experienced more anxiety than their pain-free counterparts in a case-control design (Uçar et al. 2020). Similarly, we found that the latent class with high physical and psychological complaints experienced headaches, sleep difficulties, irritability, as well as nervousness, and feeling low – the latter two complaints were not tested by Uçar and colleages (2020). Our study builds on this work and advances the literature by showing which health complaints co-occur.

Our findings on the co-occurrence of psychosomatic health complaints are supported by the shared vulnerability model among adolescents (Jastrowski Mano et al. 2019). The shared vulnerability model suggests that physical pain and anxiety have shared predisposing vulnerabilities, emotional responses, and maintenance factors (Jastrowski Mano et al. 2019). The most frequently reported complaint among adolescents in our study was irritability (reported among 42.7% of adolescents) and it co-occurred with different complaints across the latent classes. Among one group of adolescents, irritability predominantly (i.e., more than 10% of adolescents reported said complaint) co-occurred with sleep difficulties and dizziness and among another group it predominantly co-occurred with nervousness, headaches, sleep difficulties, and feeling low. Not only do health complaints co-occur, a systematic review found that health risk behaviours (e.g., smoking, physical inactivity, sexual risk behaviours, unhealthy diet) also co-occur and that the strongest predictor of engaging in multiple risk behaviours is low SES (Meader et al. 2016).

Our study found that household SES was associated with more health complaints, consistent with previous research (Ravens-Sieberer et al. 2009; Elgar et al. 2013). This finding can be explained by the social causation hypothesis which posits that stress and other factors associated with deprivation contribute to mental problems among children and adolescents (Reiss 2013). Domenico and Fournier (2014) further explain the association between deprivation and mental health by suggesting that basic psychological needs act as moderating factors between SES and number of health complaints. They found that when basic psychological needs are met, it negatively moderated the association between SES and the number of health complaints (Di Domenico and Fournier 2014).

Also consistent with previous research, we found that psychosomatic classes were not associated with national wealth and income inequality (Holstein et al. 2009; Ottová-Jordan et al. 2015). This indicates that adolescent health complaints are sensitive to household SES but not country-level wealth or inequality . Similar associations were reported in an earlier HBSC study on life satisfaction (Levin et al. 2011) and health complaints (Cosma et al. 2020; Dierckens et al. 2020). This suggests that household SES plays a stronger role in adolescents’ wellbeing relative to their country’s wealth or inequalities consistently up to the years 2017/2018 (i.e., before the COVID-19 pandemic).

Strengths of our study include our application of MLCA in examining classes of symptom profiles rather than the traditional monotonic approach (more is worse). Our study identified classes of symptoms that co-occur and the socioeconomic patterning of these classes. Mental health research generally follows a variable-centred approach that operationalises the structure of complex health concepts and then analyzes minute but statistically significant associations between single variables, usually involving symptoms or a measure of disability or impairment. It is purposefully atomistic in its approach and contrives a level of precision, statistical control and sometimes causal inference that are not ecologically valid. Person-centred analyses like MLCA complements this approach by describing naturally occurring classes of contextual and individual characteristics and health complaints. It portrays adolescents’ health and wellbeing in a more holistic fashion and shifts the focus of the analysis from contrived measurement-driven concepts to groups of adolescents that share certain characteristics and experiences (Magnusson and Stattin 1998). This idiographic, person-centred approach takes within-individual variations into account rather than dismissing them as measurement error. MLCA thereby creates knowledge that has greater ecological validity and practical relevance for health policy and programs including psychological practice and intervention.

As for limitations of our study, adolescents missing information on one of the FAS questions were excluded from the Ridit calculations (due to the nature of the analysis; n=16,957 out of 228,979); thus, only adolescents with complete data on household SES were included (Bross 1958; Elgar et al. 2017). Also, the measurement of household SES can be viewed as a rudimentary consumption-based measure of material wealth, different results might have emerged had we used data on household income or parental occupation or education. Such findings would help direct public health policies to focus on reducing socioeconomic inequalities in the society; as inequalities are associated with a higher prevalence of health risk behaviours, health complaints among children and adolescents (Pillas et al. 2014; Meader et al. 2016; Carrilero et al. 2021) . Developing more equitable societies means sustained investments in structural and social changes that promote neighborhood physical spaces, access to resources (e.g., health care, social care, education, healthy food, and drinking water), and fostering cultural norms to value healthy communities (Dubowitz et al. 2016).

Future research may wish to evaluate the co-occurrence of heath complaints with positive health behaviours and experiences (Petersen et al. 2019). For example, Elvin et al. (2021) identified latent profiles for irritability with positive well-being characteristics. The authors found that adolescents with high irritability had low emotional self-regulation and those with moderate irritability had low behavioural control. Both groups of adolescents had higher depression and anxiety symptoms as well when compared with their low irritability peers. Adolescents with low irritability were associated with high positive well-being characteristics. As such, the authors suggested promoting programs that emphasize positive functioning among adolescents by focusing on developing emotional self-regulation, behavioural control, and pro-social functioning among other positive well-being characteristics (Elvin et al. 2021). Research and policies benefit from such findings as they provide holistic understandings of adolescents’ co-occurring health related behaviours (both positive and negative) in the aim of leveraging these findings and positive well-being characteristics towards healthier policies and programs for adolescents’ health. Practical implications of our findings suggest that improving health complaints among adolescent populations should consider their background, socio-demographics, and possibly other social and structural determinants of health.

## Conclusion

Our study presented a novel approach in evaluating physical and psychological health complaints among adolescents by grouping co-occurring complaints into latent classes and assessing the association between these latent classes of health complaints with individual and macro-level SES across adolescents in Canada and countries in Europe using multilevel analysis. We found that adolescents belong to one of three distinct latent classes of latent classes: no health complaints, psychological complaints, or physical and psychological health complaints. Among the group of adolescents with psychological complaints, irritability predominantly co-occurred with sleep difficulties and dizziness and among the group with physical and psychological health complaints, irritability predominantly co-occurred with nervousness, headaches, sleep difficulties, and feeling low. Understanding co-occurring complaints will inform future studies as to which complaints to further investigate in developmental, prospective, and causal investigations with health outcomes. Adolescents who reported lower SES also reported being in a latent class with health complaints; however, this was only significant at the household-level, not at the macro/country-level.

## References

[CR1] Asmundson GJG, Katz J (2009) Understanding the co-occurrence of anxiety disorders and chronic pain: State-of-the-art. Depress. Anxiety 10.1002/da.20600 19691031

[CR2] Bross, I. D. J. (1958). How to Use Ridit Analysis. *Biometrics.*10.2307/2527727

[CR3] Carrilero, N., Dalmau-Bueno, A., & García-Altés, A. (2021). Socioeconomic inequalities in 29 childhood diseases: evidence from a 1,500,000 children population retrospective study. *BMC Public Health.*10.1186/s12889-021-11230-9 10.1186/s12889-021-11230-9 PMC8205646 34130683

[CR4] Chzhen, Y., de Neubourg, C., Plavgo, I., & de Milliano, M. (2016). Child Poverty in the European Union: the Multiple Overlapping Deprivation Analysis Approach (EU-MODA). *Child Indic Res.*10.1007/s12187-015-9321-7

[CR5] Coley, R. L., Sims, J., Dearing, E., & Spielvogel, B. (2018). Locating Economic Risks for Adolescent Mental and Behavioral Health: Poverty and Affluence in Families, Neighborhoods, and Schools. *Child Dev.*10.1111/cdev.12771 10.1111/cdev.12771 PMC5573665 28245340

[CR6] Collins LM, Lanza ST (2010) Latent Class and Latent Tranistion Analysis With Applications in the Social, Behavioral, and Health Sciences. John Wiley & Sons, Inc., New Jersey

[CR7] Corell M, Chen Y, Friberg P (2021). Does the family affluence scale reflect actual parental earned income, level of education and occupational status? A validation study using register data in Sweden. BMC Public Heal.

[CR8] Cosma, A., Stevens, G., Martin, G., et al. (2020). Cross-National Time Trends in Adolescent Mental Well-Being From 2002 to 2018 and the Explanatory Role of Schoolwork Pressure. *J Adolesc Heal.*10.1016/j.jadohealth.2020.02.010 10.1016/j.jadohealth.2020.02.010 PMC8131201 32446609

[CR9] Currie, C., Molcho, M., Boyce, W., et al. (2008). Researching health inequalities in adolescents: The development of the Health Behaviour in School-Aged Children (HBSC) Family Affluence Scale. *Soc Sci Med.*10.1016/j.socscimed.2007.11.024 10.1016/j.socscimed.2007.11.024 18179852

[CR10] Currie C, Zanotti C, Morgan A, et al (2012) Social determinants of health and well-being among young pople. Heal Behav Sch Child Study Int Rep From 2009/2010 Surv

[CR11] Deci, E. L., & Ryan, R. M. (2000). The “what” and “why” of goal pursuits: Human needs and the self-determination of behavior. *Psychol Inq.*10.1207/S15327965PLI1104_01

[CR12] Di Domenico, S. I., & Fournier, M. A. (2014). Socioeconomic Status, Income Inequality, and Health Complaints: A Basic Psychological Needs Perspective. *Soc Indic Res.*10.1007/s11205-013-0572-8

[CR13] Dierckens, M., Weinberg, D., Huang, Y., et al. (2020). National-Level Wealth Inequality and Socioeconomic Inequality in Adolescent Mental Well-Being: A Time Series Analysis of 17 Countries. *J Adolesc Heal.*10.1016/j.jadohealth.2020.03.009 10.1016/j.jadohealth.2020.03.009 32446605

[CR14] Dorling, D., Mitchell, R., & Pearce, J. (2007). The global impact of income inequality on health by age: An observational study. *Br Med J.*10.1136/bmj.39349.507315.DE 10.1136/bmj.39349.507315.DE PMC2043415 17954512

[CR15] Dubowitz, T., Orleans, T., Nelson, C., et al. (2016). Creating healthier, more equitable communities by improving governance and policy. *Health Aff.*10.1377/hlthaff.2016.0608 10.1377/hlthaff.2016.0608 27834235

[CR16] Elgar, F. J., Craig, W., & Trites, S. J. (2013). Family dinners, communication, and mental health in Canadian adolescents. *J Adolesc Heal.*10.1016/j.jadohealth.2012.07.012 10.1016/j.jadohealth.2012.07.012 23299005

[CR17] Elgar FJ, Xie A, Pförtner T-K, et al (2017) Assessing the View From Bottom: How to Measure Socioeconomic Position and Relative Deprivation in Adolescents

[CR18] Elvin, O. M., Modecki, K. L., Finch, J., et al. (2021). Joining the pieces in childhood irritability: Distinct typologies predict conduct, depressive, and anxiety symptoms. *Behav Res Ther.*10.1016/j.brat.2020.103779 10.1016/j.brat.2020.103779 33291055

[CR19] Gariepy, G., McKinnon, B., Sentenac, M., & Elgar, F. J. (2016). Validity and Reliability of a Brief Symptom Checklist to Measure Psychological Health in School-Aged Children. *Child Indic Res.*10.1007/s12187-015-9326-2

[CR20] Hagquist, C., Due, P., Torsheim, T., & Välimaa, R. (2019). Cross-country comparisons of trends in adolescent psychosomatic symptoms - A Rasch analysis of HBSC data from four Nordic countries. *Health Qual Life Outcomes.*10.1186/s12955-019-1097-x 10.1186/s12955-019-1097-x PMC6366074 30728023

[CR21] Hammami, N., Chaurasia, A., Bigelow, P., & Leatherdale, S. T. (2019). A gender-stratified, multilevel latent class assessment of chronic disease risk behaviours’ association with BMI among youth in the COMPASS study. *Prev Med (Baltim), 126*. 10.1016/j.ypmed.2019.105758 10.1016/j.ypmed.2019.105758 31254539

[CR22] Haugland, S., & Wold, B. (2001). Subjective health complaints in adolescence - Reliability and validity of survey methods. *J Adolesc.*10.1006/jado.2000.0393 10.1006/jado.2000.0393 11676508

[CR23] Henry KL, Muthén B (2010). Multilevel Latent Class Analysis: An Application of Adolescent Smoking Typologies with Individual and Contextual Predictors. Struct Equ Model.

[CR24] Hetland J, Torsheim T, Aarø LE (2002). Subjective health complaints in adolescence: Dimensional structure and variation across gender and age. Scand J Public Health.

[CR25] Hobza, V., Hamrik, Z., Bucksch, J., & De Clercq, B. (2017). The family affluence scale as an indicator for socioeconomic status: Validation on regional income differences in the Czech Republic. *Int J Environ Res Public Health.*10.3390/ijerph14121540 10.3390/ijerph14121540 PMC5750958 29292773

[CR26] Holstein, B. E., Currie, C., Boyce, W., et al. (2009). Socio-economic inequality in multiple health complaints among adolescents: International comparative study in 37 countries. *Int J Public Health.*10.1007/s00038-009-5418-4 10.1007/s00038-009-5418-4 19639254

[CR27] Inchley J, Currie D, Budisavljevic S, et al (2020) Spotlight on adolescent health and well-being: Findings from the 2017/2018 Health Behaviour in School-aged Children (HBSC) survey in Europe and Canada. http://www.hbsc.org/publications/international/. Accessed 19 Jan 2021

[CR28] Jastrowski Mano KE, O’bryan EM, Gibler RC, Beckmann E (2019) The Co-occurrence of Pediatric Chronic Pain and Anxiety: A Theoretical Review of a Developmentally Informed Shared Vulnerability Model. Clin. J. Pain 10.1097/AJP.0000000000000763 31513056

[CR29] Lanza ST, Collins LM, Lemmon DR, Schafer JL (2007). PROC LCA : A SAS Procedure for Latent Class Analysis PROC LCA : A SAS Procedure for Latent Class Analysis. Struct Equ Model.

[CR30] Levin, K. A., Torsheim, T., Vollebergh, W., et al. (2011). National Income and Income Inequality, Family Affluence and Life Satisfaction Among 13 year Old Boys and Girls: A Multilevel Study in 35 Countries. *Soc Indic Res.*10.1007/s11205-010-9747-8 10.1007/s11205-010-9747-8 PMC3183268 21980216

[CR31] Mackenbach, J. P., & Kunst, A. E. (1997). Measuring the magnitude of socio-economic inequalities in health: An overview of available measures illustrated with two examples from Europe. *Soc Sci Med.*10.1016/S0277-9536(96)00073-1 10.1016/s0277-9536(96)00073-1 9080560

[CR32] Magnusson D, Stattin H (1998) Person-context interaction theories. In: Handbook of child psychology: Volume 1: Theoretical models of human development (5th ed.). pp 685–759

[CR33] Marmot M (2004) THE STATUS SYNDROME: How Social Standing Affects Our Health and Longevity

[CR34] Meader, N., King, K., Moe-Byrne, T., et al. (2016). A systematic review on the clustering and co-occurrence of multiple risk behaviours. *BMC Public Health.*10.1186/s12889-016-3373-6 10.1186/s12889-016-3373-6 PMC4966774 27473458

[CR35] Molcho M, Gabhainn SN, Kelleher CC (2007) Assessing the use of the Family Affluence Scale (FAS) among Irish schoolchildren. Ir Med J 17955700

[CR36] Muthén LK, Muthén B (2018) Mplus

[CR37] Ottová-Jordan V, Smith ORF, Augustine L, et al (2015) Trends in health complaints from 2002 to 2010 in 34 countries and their association with health behaviours and social context factors at individual and macro-level. Eur. J. Public Health 10.1093/eurpub/ckv033 25805796

[CR38] Patton GC, Sawyer SM, Santelli JS, et al (2016) Our future: a Lancet commission on adolescent health and wellbeing. Lancet 10.1016/S0140-6736(16)00579-1 PMC5832967 27174304

[CR39] Petersen KJ, Qualter P, Humphrey N (2019) The application of latent class analysis for investigating population child mental health: A systematic review. Front. Psychol. 10 10.3389/fpsyg.2019.01214 PMC6548989 31191405

[CR40] Pillas D, Marmot M, Naicker K, et al (2014) Social inequalities in early childhood health and development: A European-wide systematic review. Pediatr. Res. 10.1038/pr.2014.122 25122581

[CR41] Potrebny, T., Wiium, N., & Lundegård, M. M. I. (2017). Temporal trends in adolescents’ self-reported psychosomatic health complaints from 1980-2016: A systematic review and meta-analysis. *PLoS One, 12*. 10.1371/journal.pone.0188374 10.1371/journal.pone.0188374 PMC5705135 29182644

[CR42] Poznanski, B., Cornacchio, D., Coxe, S., et al. (2018). The Link Between Anxiety Severity and Irritability Among Anxious Youth: Evaluating the Mediating Role of Sleep Problems. *Child Psychiatry Hum Dev.*10.1007/s10578-017-0769-1 10.1007/s10578-017-0769-1 29222620

[CR43] Ravens-Sieberer, U., Erhart, M., Torsheim, T., et al. (2008). An international scoring system for self-reported health complaints in adolescents. *Eur J Public Health.*10.1093/eurpub/ckn001 10.1093/eurpub/ckn001 18252752

[CR44] Ravens-Sieberer, U., Torsheim, T., Hetland, J., et al. (2009). Subjective health, symptom load and quality of life of children and adolescents in Europe. *Int J Public Health.*10.1007/s00038-009-5406-8 10.1007/s00038-009-5406-8 19639258

[CR45] Reiss F (2013). Socioeconomic inequalities and mental health problems in children and adolescents: A systematic review. Soc. Sci. Med..

[CR46] Roberts C, Currie C, Samdal O, et al (2007) Measuring the health and health behaviours of adolescents through cross-national survey research: Recent developments in the Health Behaviour in School-aged Children (HBSC) study. In: Journal of Public Health

[CR47] Roberts, C., Freeman, J., Samdal, O., et al. (2009). The Health Behaviour in School-aged Children (HBSC) study: Methodological developments and current tensions. *Int J Public Health.*10.1007/s00038-009-5405-9 10.1007/s00038-009-5405-9 PMC2732766 19639259

[CR48] Solt, F. (2016). The Standardized World Income Inequality Database*. *Soc Sci Q.*10.1111/ssqu.12295

[CR49] Stata Press (2019) Stata Statistical Software: Release 16. In: StataCorp LLC

[CR50] Swift, R. (2011). The relationship between health and GDP in OECD countries in the very long run. *Health Econ.*10.1002/hec.1590 10.1002/hec.1590 20217835

[CR51] The World Bank (2018). GDP per capita, Atlas method (current US$).

[CR52] Uçar, H. N., Tekin, U., & Tekin, E. (2020). Irritability and its relationships with psychological symptoms in adolescents with migraine: a case-control study. *Neurol Sci.*10.1007/s10072-020-04331-7 10.1007/s10072-020-04331-7 32212010

[CR53] Vaičiūnas, T., & Šmigelskas, K. (2019). The role of school-related well-being for adolescent subjective health complaints. *Int J Environ Res Public Health.*10.3390/ijerph16091577 10.3390/ijerph16091577 PMC6540129 31064078

[CR54] Ward, J. L., & Viner, R. M. (2017). The impact of income inequality and national wealth on child and adolescent mortality in low and middle-income countries. *BMC Public Health.*10.1186/s12889-017-4310-z 10.1186/s12889-017-4310-z PMC5425964 28490327

[CR55] Wilkinson RG, Pickett KE (2009). The spirit level: Why more equal societies almost always do better.

